# Low Maternal Serum 25‐Hydroxyvitamin D Concentration Is Associated With Postpartum Hemorrhage: A Retrospective Observational Study

**DOI:** 10.3389/fendo.2022.816480

**Published:** 2022-03-17

**Authors:** Wei-Jiun Li, Kuo-Hu Chen, Lee-Wen Huang, Yieh-Loong Tsai, Kok-Min Seow

**Affiliations:** ^1^Department of Obstetrics and Gynecology, Shin-Kong Wu Ho-Su Memorial Hospital, Taipei, Taiwan; ^2^Department of Obstetrics and Gynecology, Taipei Tzu-Chi Hospital, The Buddhist Tzu-Chi Medical Foundation, Taipei, Taiwan; ^3^School of Medicine, Buddhist Tzu-Chi University, Hualien, Taiwan; ^4^College of Medicine, Fu-Jen Catholic University, New Taipei City, Taiwan; ^5^Department of Obstetrics and Gynecology, National Yang Ming Chiao Tung University, Taipei, Taiwan

**Keywords:** postpartum hemorrhage and 25-hydroxyvitamin D concentration pregnancy, uterine atony, 25(OH)D, vitamin D, postpartum hemorrhage

## Abstract

**Objective:**

The primary aim of this study is to investigate the relationship between vitamin D serum level and the incidence of postpartum hemorrhage (PPH). The secondary objective is to determine the relative risk of low vitamin D associated with PPH.

**Methods:**

This was a retrospective observational study. A total of 600 women who had delivered their babies in a single tertiary teaching hospital were enrolled. Serum blood test for 25(OH)D was performed at 35 + 0 to 36 + 6 weeks of pregnancy to measure vitamin D. A 25(OH)D level < 20 ng/mL was defined as vitamin D deficient, and a level 21–29 ng/mL as insufficient.

**Results:**

Vitamin D levels were deficient in 145 (24.1%) and insufficient in 254 (42.3%) of the women tested. Women with deficient and insufficient vitamin D levels were significantly younger than those with sufficient vitamin D levels (p < 0.001). The overall rates of PPH in the deficient and insufficient groups were 6.9% (10/145) and 6.7% (17/254), respectively, and were significantly higher than the rate of the normal vitamin D group (1.5%, p = 0.009). Women with sufficient vitamin D levels had significantly higher hemoglobin levels than those with low vitamin D levels. Higher vitamin D levels were associated with a significantly low risk of PPH (AOR: 0.93, CI: 0.89–0.98, p = 0.006).

**Conclusion:**

Our results suggest that a low vitamin D level is a risk factor for PPH. Low vitamin D also related to high risk of low hemoglobin before delivery. Thus, antepartum care should include vitamin D supplements for all women if possible.

## Introduction

Postpartum hemorrhage (PPH), defined as estimated blood loss of at least 500 mL following a vaginal delivery or 1000 mL after a cesarean delivery within 24 h postpartum, is one of the most common causes of maternal mortality worldwide (1), 2) (2).PPH may cause sudden massive life-threatening hemorrhage (3) and maternal deaths (1). The most common cause of PPH is uterine atony, which contributes to up to 80% of cases (4). Although many risk factors for uterine atony have been reported (advanced maternal age, multiple gestations, prior hemorrhage, prolonged labor, obesity, White or Hispanic race/ethnicity, polyhydramnios, preeclampsia, anemia, and chorioamnionitis infection), the reasons and risk factors for PPH remain controversial (5).

Vitamin D is a fat-soluble vitamin produced in the body through the interaction of sunlight with 7-dehydrocholesterol (6), and it is essential to maintain human health (7, 8). There are two main isoforms of vitamin D, namely, D3 (cholecalciferol) and D2 (ergocalciferol) (9). Vitamin D3 is derived from ultraviolet-B and is found in foods such as fatty fish, cod liver oil, and egg yolk. Vitamin D2 is derived from the intake of fungal sources such as mushrooms and yeast. Vitamin D deficiency may lead to softening of bones, osteomalacia, and rickets as it plays an important role in bone and calcium (mineral) homeostasis (10, 11). Furthermore, several observational studies have shown that vitamin D deficiency may induce various nonskeletal diseases such as cardiovascular (12, 13), and metabolic diseases (14), cancer (15), autoimmune (16), and neurological disorders (17). Therefore, maintenance of normal vitamin D levels is important for everyone. Nevertheless, vitamin D deficiency is a common health problem worldwide. It has been reported that 30%–50% of the population in the United States, Canada, Europe, Australia, New Zealand, and Asia are vitamin D deficient (18). A study from a Mediterranean country reported the prevalence of vitamin D inadequacy as 86.4% in males and 82.3% in females, with a cut-off value of 25(OH)D < 30 ng/mL (19). Some studies also demonstrated that vitamin D deficiency was more prevalent among women and younger age groups (19, 20). Vitamin D is converted to 25-hydroxyvitamin D (25[OH]D) in the liver and has a half-life of approximately 2 to 3 weeks (21). Serum 25(OH)D is used to assess the status of vitamin D because it best reflects its long half-life in circulation and the supply from different sources, such as that produced cutaneously and that obtained from foods and supplements. In an adult, serum 25(OH)D levels < 20 ng/mL are defined as deficient, 20–30 ng/mL as insufficient, and above 30 ng/mL as sufficient (6–8).

Evidence indicates that low vitamin D levels during pregnancy lead to an increased rate of low birth weight in neonates (22–24). Vitamin D supplementation increased mean birth weight by 58.33 g (95% CI: 18.88–97.98 g) and reduced the risk of small for gestational age (SGA) with a pooled risk ratio of 0.60 (95% CI: 0.40–0.90) in a meta-analysis (25). Vitamin D deficiency is also a risk factor for multiple maternal disorders such as preeclampsia, insulin resistance, gestational diabetes mellitus (GDM), and a higher risk of primary cesarean delivery (26–29).

Supplementation with vitamin D alone during pregnancy may reduce the risk of preeclampsia and GDM (30). A lower serum level of vitamin D during pregnancy is one of the risks factors for PPH and uterine atony (30). Vitamin D supplementation reportedly may reduce the risk of severe PPH according to one study (30). However, this result is based on findings from only a single trial, and it was an unexpected finding not previously documented.

In this study, we assessed the effects of low vitamin D in association with PPH and uterine atony. We report the primary outcome as the relationship between low serum levels of vitamin D and the incidence of PPH and the secondary outcome as the relative risk of low vitamin D associated with PPH.

## Materials and Methods

For this retrospective observational study, we reviewed the records for all pregnant women who delivered a single baby at Shin Kong Wu Ho-Su Memorial Hospital, Taipei, Taiwan, between October 2019 and March 2021. All women received regular prenatal care from 8 + 0 weeks and delivered their babies at term. The inclusion criteria for study subjects were singleton pregnancy and good health without preeclampsia, immune disease, placenta abruption, etc. The protocol was reviewed and approved by the institutional review board of Shin Kong Wu Ho-Su Memorial Hospital, Taipei, Taiwan. The first and corresponding authors reviewed the medical records.

Vitamin D deficiency was defined as <20 ng/mL of 25(OH)D, and insufficiency as 21–29 ng/mL (22–25). The first 25(OH)D blood measurement occurred at 12 + 0 to 13 + 6 weeks of gestational age. If 25(OH)D was <30 ng/mL, a vitamin D supplement of 2000 IU daily was suggested. A second blood test for 25(OH)D was performed at 35 + 0 to 36 + 6 weeks.

Patient demographic and clinical data were obtained by manual chart review. Maternal demographic and clinical characteristics included maternal age, body weight, delivery mode, fetal body weight, vitamin D levels, and blood loss. Hemoglobin levels were also recorded before and after delivery. The women were divided into three groups according to vitamin D serum levels at 35 + 0 to 36 + 6 weeks. Group 1 included women with serum 25(OH)D levels ≤ 20 ng/mL, group 2 included women with levels of 20–30 ng/mL, and group 3 included women with levels ≥ 30 ng/mL. The pregnancy and maternal outcomes were compared between groups. Preterm delivery was defined as <37 weeks at delivery, and low birth weight was defined as full-term < 2500 g or preterm < 10th percentile of the population. Blood loss > 500 mL following a vaginal delivery or more than 1000 mL following a cesarean delivery was considered PPH.

### Assessment of 25-Hydroxyvitamin D (25-OHD) Circulating Levels

A blood sample was drawn from the antecubital vein of each participant at 12 + 0 to 13 + 6 and 35 + 0 to 36 + 6 weeks gestational age. Serum samples were tested using an automated chemiluminescence micro-particle immunoassay (CMIA) kit (ARCHITECT; Abbott Laboratories, Abbott Park, IL, USA) to quantify 25-OHD.

### Statistical Analyses

Data are presented as mean ± standard deviation. Categorical variables are presented as frequencies and valid percentages. The Student’s t-test was used to assess the difference in means between groups, and chi-square and Fisher’s exact tests were used where appropriate. Longitudinal data were compared using the analysis of variance (ANOVA) for three groups. We used multivariable Poisson regression models to examine the correlation between maternal vitamin D and maternal and fetal outcomes, adjusting for maternal age, gestational age, delivery methods, body mass index (BMI), platelet count, and hemoglobin. The results are presented as the adjusted odds ratio (AOR) with a 95% confidence interval (CI). A P value of <0.05 was considered the threshold of significance. The statistical analysis was conducted using SPSS Version 26 software (IBM Corp., Armonk, NY, USA).

## Results

**Figure 1:**
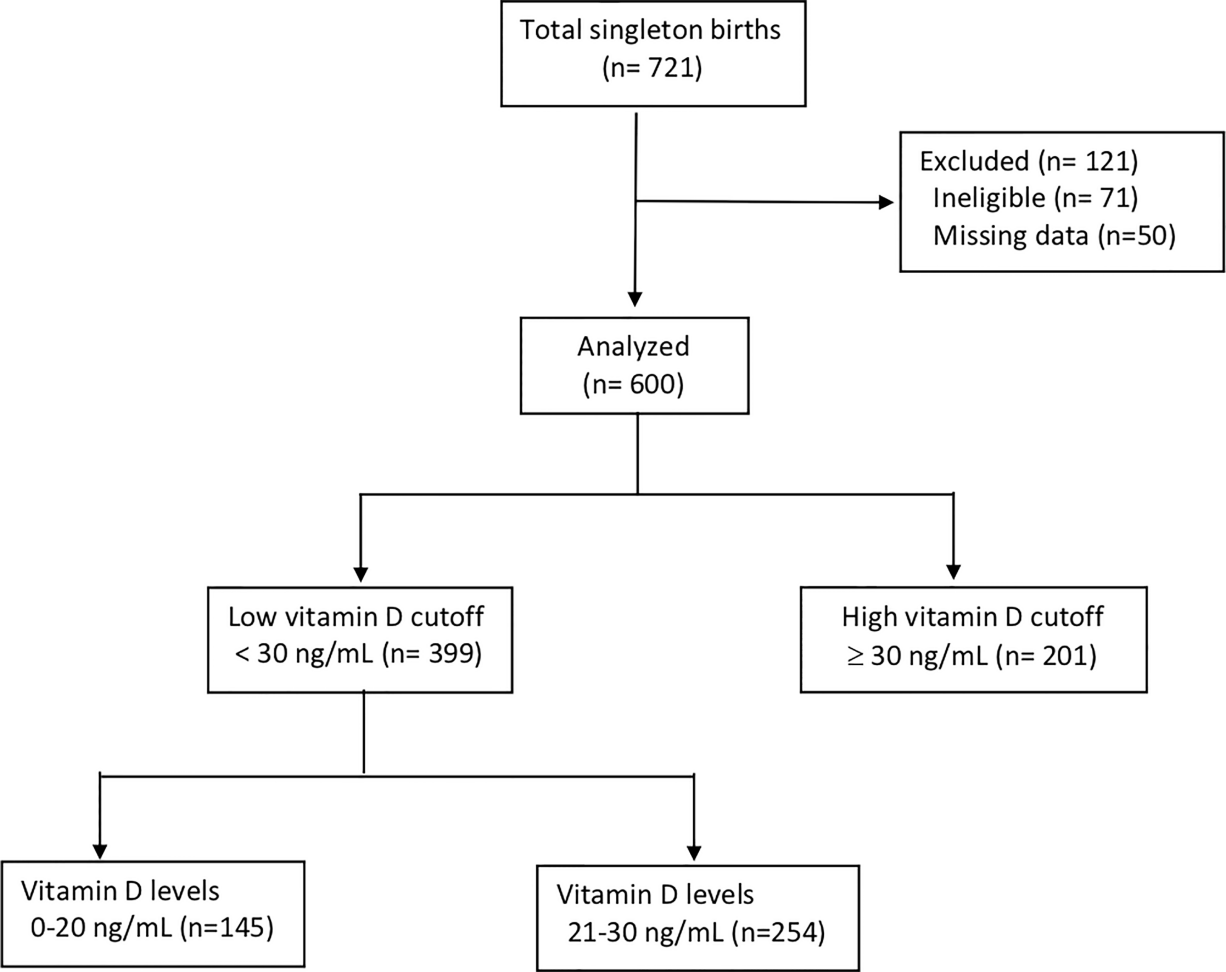
Study cohort.

Of the 721 women available for this retrospective observational study, 600 (83.2%) met the inclusion criteria (**Figure 1**). More than half of the women (66.5%) had low vitamin D levels (<30 ng/mL). Of these, vitamin D levels were deficient (<20 ng/mL) in 145 (24.1%) and insufficient in 254 (42.3%). Only 201 (33.5%) women had vitamin D levels > 30 ng/mL. Women with deficient and insufficient vitamin D levels were significantly younger than those with sufficient levels. The gestational age at delivery and delivery methods were not significantly different in the three groups. Only approximately 30% of the women in this study took vitamin D during pregnancy even though it was recommended. Women with compliance to have vitamin D supplement may have higher vitamin D even though there was no significant different. For those women who have low vitamin D level at 12 + 0 to 13 + 6 weeks and with compliance to have vitamin D supplement with 2000IU per day, eighty percent of them may have 25(OH)D level >30 ng/mL (data not shown). The fetal body weights and estimated blood loss were not significantly different in the three groups. However, the hemoglobin level was significantly higher in women with sufficient vitamin D levels than in those with insufficient and deficient levels (p < 0.001). The estimated blood loss was not significantly different in the three groups (**Table 1**).

**Table 1:** Demographic and clinical characteristic comparison based on 25(OH)D levels.

Variables	Group ≤20 ng/mL n = 145			Group 2 21–30 ng/mL n = 254	Group 3 ≥30 ng/mL n = 201	P
**Age**		31.8 ± 4.8	32.6 ± 4.5		34.7 ± 4.5	**<0.001**
**BMI (kg/m^2^)**		26.8 ± 3.7	27 ± 3.9		26.5 ± 3.4	0.184
**Parity**		0.97	1.2		1.1	0.111
**GA at delivery**		38.6 ± 0.98	38.3 ± 1.1		38.5 ± 0.5	0.128
**Delivery methods**						
** NSD**		54 (37.2%)	103 (40.6%)		73 (36%)	0.832
** VED**		35 (24.1%)	65 (25.6%)		54 (27.1%)	0.277
** C/S**		55 (37.9%)	86 (33.9%)		73 (36%)	0.187
** VBAC**		1 (0.7%)	0 (0%)		1 (0.5%)	0.853
**Vitamin D sup.**		40 (27.6%)	76 (29.9%)		65 (32.4%)	0.603
**FBW (gm)**		3078 ± 372	3087 ± 371		3076 ± 361	0.941
**EBL (cc)**		472 ± 437	465 ± 457		464 ± 440	0.984
**Hb (gm/dl)**		11.3 ± 1.4	11.9 ± 1.2		12.1 ± 1.3	**<0.001**
**Platelets (×10^3^)**		246 ± 65	235 ± 61		231 ± 63	0.075
**PT (sec)**		10 ± 0.4	10.1 ± 0.8		10 ± 0.5	0.102

BMI, body mass index; C/S, cesarean section; EBL, estimated blood loss; FBW, fetal body weight; GA, gestational age; Hb, hemoglobin; NSD, normal spontaneous delivery; PT, prothrombin time; VBAC, vaginal birth after cesarean section; VED, vacuum extraction delivery.

P < 0.05 is significant. Bold values mean that: P< 0.05.

Differences in the rates of adverse maternal outcomes associated with vitamin D levels are shown in **Table 2**. The rate of GDM was not significantly different between the three groups. There was an increasing trend of pregnancy-induced hypertension in groups 1 and 2 compared to group 3, but it was not significantly different. However, the rate of PPH was significantly higher in both groups 1 and 2 compared to group 3. The fetal body weight (<2500 g) and preterm delivery < 37 weeks were also not significantly different between groups.

**Table 2:** Maternal outcomes according to 25(OH)D levels.

	Group 1 ≤20 ng/mL n = 145	Group 2 21–30 ng/mL n = 254	Group 3 ≥30 ng/mL n = 201	P
**GDM**	8 (5.5%)	24 (9.5%)	22 (11%)	0.185
**PIH **	3 (2.1%)	12 (4.7%)	4 (1.9%)	0.128
**PPH**	10 (6.9%)	17 (6.7%)	3 (1.5%)	**0.009**
**FBW**				
** ≥2500 g**	139 (95.9%)	234 (92.1%)	190 (94.5%)	0.338
** <2500**	6 (4.1%)	20 (7.9%)	11 (5.5%)	0.345
**GA**				
** ≥ 37 wks**	141 (97.2%)	239 (94.1%)	194 (96.5%)	0.312
** <37 wks**	4 (2.8%)	15 (5.9%)	7 (3.5%)	0.432

FBW, fetal body weight; GA, gestational age; GDM, gestational diabetes; PIH, pregnancy-induced hypertension; PPH, postpartum hemorrhage.

P < 0.05 is significant. Bold values mean that: P< 0.05.

**Table 3** shows the relationship between PPH and possible potential risk factors. Higher vitamin D levels were associated with a significantly low risk of PPH (AOR: 0.93, CI: 0.89–0.98, p = 0.006). In this study, *per* I ng/mL increase of vitamin D led to 7% lower likelihood of developing PPH. No relationship with PPH was found for age, hemoglobin, BMI, and platelet count.

**Table 3:** Logistic regression analysis of BMI, HB, platelets, and vitamin D on PPH.

Variables	Adjusted OR	95% CI	P
**Age**	1.01	0.98–1.22	0.190
**BMI**	0.94	0.85–1.05	0.291
**Hb**	0.83	0.61–1.12	0.291
**Platelets**	0.99	0.99–1.12	0.416
**Vitamin D**	0.93	0.89–0.98	**0.006**

BMI, body mass index; CI, confidence interval; Hb, hemoglobin; OR, odds ratio; PPH, postpartum hemorrhage.

P < 0.05 is significant. Bold values mean that: P< 0.05.

## Discussion

Our study demonstrated that vitamin D levels were deficient (<20 ng/mL) in 145 (24.1%) and insufficient in 254 (42.3%) women, compatible with previous report that 22.9% of Taiwan women with a vitamin levels < 20 ng/mL (31). This study is the first reported in the literature to assess the effect of vitamin D on PPH. In this retrospective study, we found that the rate of PPH was significantly higher in low vitamin D groups (25[OH]D levels < 30 ng/mL) compared with a normal vitamin D group (25(OH)D levels ≥ 30 ng/mL). On the basis of our results, women with a low vitamin D level had a four- to fivefold risk of PPH (6.7%–6.9% vs. 1.5%, p = 0.009) compared to those with vitamin D levels > 30 ng/mL. Therefore, vitamin D supplements should be suggested for all women during pregnancy.

Older pregnant women were found to have higher levels of vitamin D in this study (p < 0.001), which may be due to their financial ability to buy vitamin D because vitamin D is expensive in Taiwan. Furthermore, older women may be more cognizant regarding good fetal health when pregnant (32). Therefore, vitamin D supplements appear to be popular among older pregnant women. Other factors related to low vitamin D are ethnicity, sunlight exposure, dark skin and obesity should also be considered. However, we did not collect pregnant women with difference races and measurement the time of sunlight exposure in this study and this may be the limitation in the study.

This study also found that women with vitamin D levels > 30 ng/mL may have a significantly higher hemoglobin level than those with levels < 30 ng/mL. This finding in pregnant women has never been reported in the literature. The effect of vitamin D on hematopoiesis is largely unknown. A previous study reported that a low vitamin D level was associated with an increased risk of anemia (33). Several studies reported that vitamin D supplementation could improve anemia by increasing erythropoietin production and erythropoietin receptor expression, reducing the secretion of proinflammatory mediators, and enhancing erythropoietin sensitivity (34, 35). Furthermore, some studies found that vitamin D deficiency may increase the risk of anemia in chronic kidney disease (36, 37). Li et al. (35) reported the prevalence of anemia as high as 79.5% (Hb < 120 g/L) in a severe vitamin D-deficient group (25[OH]D levels < 30 nmol/L or 12 ng/mL). Sim et al. (38) also reported that anemia was present in 49% of 25-hydroxyvitamin D-deficient subjects compared with 36% with normal 25-hydroxyvitamin D levels. However, Kucukay et al. found that vitamin D supplements may significantly lower platelet count and mean platelet volume levels, with no significant change in hemoglobin level (39). We did not find any difference in platelet count and prothrombin time between groups. Furthermore, severe prenatal anemia was reported may increase PPH risk (40). Therefore, it is important to recommend vitamin D supplement in pregnant women to avoid antepartum anemia.

The most important finding in this study is that vitamin D deficiency was significantly related to PPH. Women with vitamin D deficiency or insufficiency may have a substantially higher risk of PPH. Only one previous study reported that a low vitamin D level was strongly related to uterine atony and increased risk of PPH (30), comparable to our study. However, this previous study had a small sample size. Vitamin D is essential for maintaining the structure and function of the musculoskeletal system (41). Several studies correlate serum vitamin D levels with muscle cell contractility, muscle strength, and postural stability (13, 42). Deficiency or insufficiency of 25(OH)D has been related to proximal muscle weakness and gait disturbance, as well as poor uterine contraction, leading to PPH (41). The mechanism of vitamin D in the regulation of uterine smooth muscle contraction is still unknown. In women with preterm labor, vitamin D may decrease the risk of preterm delivery by decreasing inflammation-induced cytokines and contractile-associated factors in uterine myometrial smooth muscle cells *via* the NF-κB pathway (43). However, there are many other validated risk factors for PPH, such as placental abruption, history of PPH, prolonged labor, use of forceps or vacuum assisted and etc.

Vitamin D deficiency is a risk factor for multiple maternal disorders such as preeclampsia, GDM, SGA, and others (7, 22, 24, 30). However, we did not find any significant differences in GDM and SGA in this study possibly due to the small sample size. There was a trend toward an increased rate of pregnancy-induced hypertension in low vitamin D groups compared with the normal vitamin D group, but it was not statistically significant. A larger sample size study may be needed in the future to determine any true association.

There are several limitations to this study. First, it was a retrospective and not a randomized study. Second, the sample size of 600 in this study is small, but it is unique in that it compares the outcomes of women with normal and low vitamin D levels for PPH. Third, there are many other risk factors for PPH that may be more important than vitamin D. However, multivariate regression analysis in this study determined that a *per* I ng/mL increase of vitamin D led to 7% lower likelihood of developing PPH, which has never been reported previously. A larger, multicenter, prospective study should be performed to verify these findings.

In conclusion, our small study revealed that a low vitamin D level is a risk factor for PPH. Low vitamin D also related to high risk of low hemoglobin before delivery. Therefore, it is strongly suggested that all women should be advised to take vitamin D supplements during pregnancy.

## Data Availability Statement

The original contributions presented in the study are included in the article/supplementary material. Further inquiries can be directed to the corresponding author.

## Ethics Statement

The studies involving human participants were reviewed and approved by Shin-Kong Wu Ho-Su Memorial Hospital. Written informed consent for participation was not required for this study in accordance with the national legislation and the institutional requirements.

## Author Contributions

All authors listed have made a substantial, direct, and intellectual contribution to the work and approved it for publication.

## Funding

This study was supported by grants from Shin Kong Wu Ho-Su Memorial Hospital, Taipei, Taiwan (grant no. 2022SKHADR020).

## Conflict of Interest

The authors declare that the research was conducted in the absence of any commercial or financial relationships that could be construed as a potential conflict of interest.

## Publisher’s Note

All claims expressed in this article are solely those of the authors and do not necessarily represent those of their affiliated organizations, or those of the publisher, the editors and the reviewers. Any product that may be evaluated in this article, or claim that may be made by its manufacturer, is not guaranteed or endorsed by the publisher.
